# A Novel Autologous Micrografts Technology in Combination with Negative Pressure Wound Therapy (NPWT) for Quick Granulation Tissue Formation in Chronic/Refractory Ulcer

**DOI:** 10.3390/healthcare8040513

**Published:** 2020-11-25

**Authors:** Satoshi Takagi, Takuto Oyama, Shiro Jimi, Arman Saparov, Hiroyuki Ohjimi

**Affiliations:** 1 Department of Plastic, Reconstructive and Aesthetic Surgery, Faculty of Medicine, Fukuoka University, Fukuoka 814-0180, Japan; oyt1974@yahoo.co.jp (T.O.); ohjimi@fukuoka-u.ac.jp (H.O.); 2 Central Lab for Pathology and Morphology, Faculty of Medicine, Fukuoka University, Fukuoka 814-0180, Japan; sjimi@fukuoka-u.ac.jp; 3 Department of Medicine, School of Medicine, Nazarbayev University, Nur-Sultan 010000, Kazakhstan; asaparov@nu.edu.kz

**Keywords:** regenerative medicine, chronic wounds, micrografts

## Abstract

Negative pressure wound therapy (NPWT) has been commonly used over the years for a wide range of chronic/refractory lesions. Alternatively, autologous micrografting technology is recently becoming a powerful modality for initiating wound healing. The case presented is of a patient with a lower leg ulcer that had responded poorly to NPWT alone for three weeks. Consequently, the patient was put on a combination therapy of NPWT and micrografting. After injection of a dermal tissue micrografts suspension into the entire wound bed, NPWT was performed successively for two weeks, resulting in fresh granulation tissue formation. Thereafter, the autologous skin graft was taken well. This case study indicates that for a chronic/refractory ulcer patient with poor NPWT outcome, combination therapy using micrografting treatment and NPWT could rapidly initiate and enhance granulation tissue formation, creating a favorable bedding for subsequent skin grafting.

## 1. Introduction

Negative pressure wound therapy (NPWT), which has been established since the 1990s, is an effective technology for wound management [1,2]. The therapy is less invasive for patients with intractable wounds, and applicable for a wide range of lesions: acute or subacute ulcers, such as massive avulsion injury and open fracture of the limb, to chronic wounds, including decubitus, venous stasis and diabetic foot ulcers [1]. The mode of action of NPWT facilitates the removal of excessive exudate or infectious discharge and the reduction of edema, and stimulates the granulation tissue formation [1]. Wound healing is a successive multistage process that involves inflammation, cell proliferation and tissue remodeling. If exacerbating factors, including microbial biofilms, necrotic tissue, wound infection and impaired circulation, are present, the cascade could derail and arrest the wound at its chronic phase. Recent documentation shows that NPWT therapy improves granulation tissue status by inducing an increase in blood perfusion and vascular endothelial growth factor (VEGF) secretion in the lesion sites [2]. It also has been demonstrated that NPWT regulates inflammatory cytokines and contributes to positive effects on a molecular biological level of the wound healing [3]. The findings denote the crucial importance of cellular reactivation in the wound for progressing the wound healing process.

Among the many clinically available technologies for wound treatments, the micrograft technique is a method using the patients’ own healthy tissue grafted onto their lesions. This is an old technique but comprises a unique medical concept. The Meek micrografting method [4], regarded as a modern micrografting technique, is adopted for extensive burn wounds, but the tissue size remains relatively large, by which the coverage area is limited in lesions [5]. Today, the tissue graft size has become even more important for inducing subsequent cellular reactions derived from the grafted tissue. In consideration of the graft size, a medical device named Rigeneracons (Human Brain Wave Srl, Torino, Italy) provides a mechanical production of double-digit micron autologous micrografts in size, which enables coverage of the entire wound area without any enzymatic digestions, allowing cellular viability to be kept at higher levels [6]. Recent studies with Rigeneracons [7,8] indicate that wound healing in mice is accelerated, and transforming growth factor-beta1 (TGF-β1) expression was selectively upregulated in granulation tissue accompanied by matured collagen matrixes even in the early phase. In addition, α-smooth muscle actin expressing myofibroblasts and neovascularization abundantly appeared in granulation tissue.

Looking back at its history, this particular micrografts technology started with hair-containing punched tissues [9], and since then, different tissues have been subjected: skin [10], dermis [11], fat [12], periosteum [13], cartilage [14], bone [15], and atrial appendage [16]. The grafts may contain by themselves resident cells, progenitor cells, as well as various tissue factors including cytokines and growth factors. This micrografts technique has been clinically applied for different pathologic lesions, such as chronic ulcers [17,18,19], exaggerated scars [20] and wound opening [10,21]. Riccio et al. (2019) [22] recently performed a multicenter clinical analysis using the micrograft technique in complicated traumatic ulcers with large loss of skin and soft tissue in the lower limbs of 70 patients, resulting in stimulation of skin regeneration with less scarring. The aim of reconstructive treatment is the morphological and functional restoration. The autologous micrografts technique utilizes a new concept in regenerative medicine for severe traumatic wounds.

In this brief communication, we report the use and beneficial effects of the Rigenera autologous micrografts technology in combination with NPWT for a refractory ulcer that improved dramatically.

## 2. Materials and Methods

A 20-year-old man fell down while riding a motorcycle and was taken to a critical care center. His consciousness was clear, no damage to the skin on the body surface was observed, and he only complained of pain and swelling in his right thigh. As a result of the medical examination, he was diagnosed as having a right femoral shaft fracture surrounded by vascular injury. On the same day, an external fixation device was applied to retain the fracture site, and the damaged superficial femoral artery was repaired. Six hours later, severe pain and swelling appeared in the right lower leg. When the intra-fascial tissue pressure was measured on the anterior and posterior surfaces of the lower leg, both of which were 80 mmHg, the patient was diagnosed with compartment syndrome and an emergency relief incision for dilatation was performed. The postoperative course was uneventful, and on the 8th day, he was introduced to us due to the unclosed wound. When transferred to our department, the patient was subjected to NPWT (−125 mmHg) alone for the first 3 weeks, and then underwent combined therapy with the Rigenera-obtained micrografts. 

**Figure 1:**
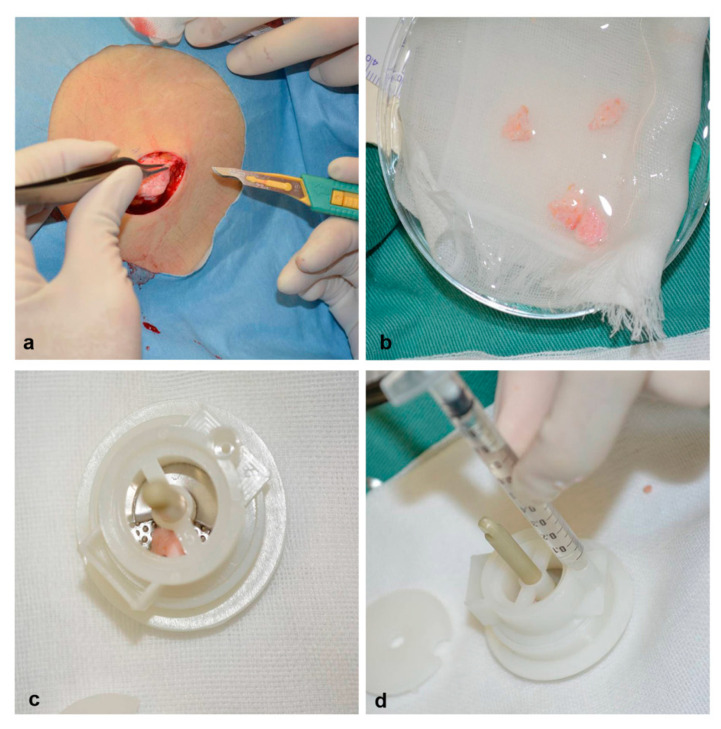
In order to use the Rigeneracons device, a small sample autologous tissue is collected from the outer part of the patient’s thigh (**a**). The dermal component of skin is selected (**b**). The tissue sample along with sterile saline solution are inserted inside the device, later activated for 2 min (**c**). The tissue is disaggregated into micrografts which are suspended within the sterile saline solution and collected with a syringe from the reservoir of the device, ready to be grafted on the patient (**d**).

In preparing the micrografts solution, a small dermal tissue sample (10 × 20 mm) was harvested from the outer part of the patient’s left thigh, which was immediately disaggregated using the Rigenera machine (Human Brain Wave Srl) as described in Figure 1. In our protocol, a total of 2.5 mL of micrografts suspension was injected several millimeters in depth around and over the wound surface. Subsequently, NPWT was applied at −125 mmHg on the same day and successively applied for 2 weeks thereafter.

## 3. Results

**Figure 2:**
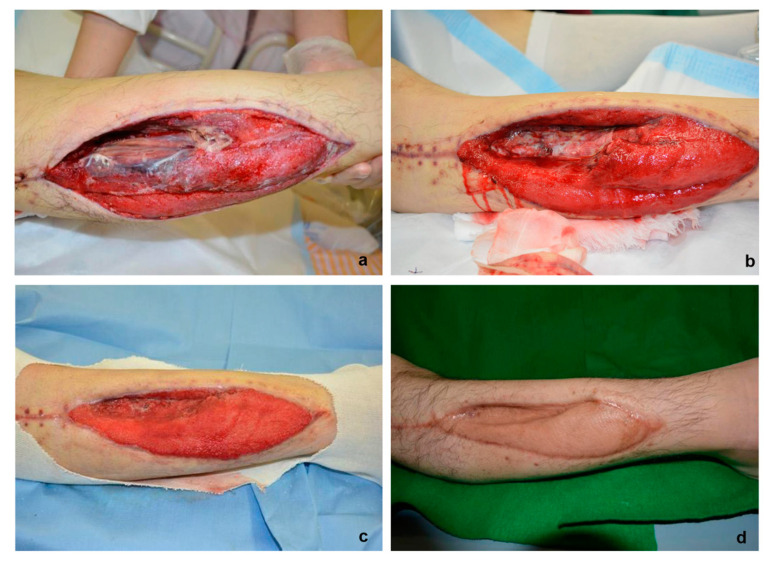
Eight days after emergency incision for the relief of lower leg compartment syndrome. The wound reached the interosseous membrane, and necrotic tissue was observed in its vicinity (**a**). Three weeks after the wound management with negative pressure wound therapy (NPWT). Still being deeply graved, the wound was complemented with micrografts treatment using Rigenera technology (**b**). In just two weeks, the wound was covered with good granulation, and meshed skin graft was performed (**c**). Six months later from the skin graft we observed 100% of graft intake (**d**).

At the first visit to our department, the tissue defect on the outer side of the patient’s right leg was 8 × 20 cm in size, forming a dent with a depth reaching the interosseous membrane (Figure 2a). Necrotic tissue was also observed in its vicinity. Wound management was started using NPWT (−125 mmHg), which led to unsatisfactory results and thin granulation tissue in the recess even after 3 weeks (Figure 2b). Therefore, our team decided to use the Rigenera micrografts technology in order to speed up the wound healing process. The micrografts suspension was injected into the ulcer floor, followed by NPWT under the same conditions (−125 mmHg). After 2 weeks, the wound was markedly covered with excellent granulation tissue (Figure 2c). Lastly, to resurface the wound, we used meshed skin grafting as the wound bed was adequate and ready; the intake rate was 100% and no relapse was seen even after 6 months (Figure 2d). 

## 4. Discussion

The present study demonstrated that in the case of a patient with chronic/refractory ulcers resulting in NPWT failure, a combination therapy of micrografts treatment and NPWT could initiate and enhance granulation tissue formation, creating a favorable bedding for subsequent skin grafting.

This micrografting technique has been used for different types of tissue injuries, and the established method, the Rigenera technology, [5] utilizes a certified medical device designed to disaggregate in only 2 min autologous grafts. In vitro studies have shown that mechanically obtained micrografts are enriched with mesenchymal progenitor cells, growth factors, and extracellular matrix components [19,23]. The technology has been successfully applied in different clinical contexts: dentistry field [13], dermatology for androgenetic alopecia [9,24], traumatology for chondropathy [14], osteonecrosis [15], and cardiology field for cardiac ischemia [16]. Our present result demonstrated that micrografts helped a severe intractable wound to generate qualified granulation tissue. Taken together, these findings signify that micrografts can enhance potential biological activity to a higher level when faced with tissue injury, which can also lead to wound healing and regeneration in different tissues. 

It is known that NPWT provides strong support in pushing the chronic wound forward and getting it through the wound healing cascade. In the wound repair process, a wide variety of growth factors or cytokines are involved, manifesting biological roles which include TGF-β1, platelet-derived growth factor, connective tissue growth factor, epidermal growth factor and VEGF [25]. They are all crucial factors for wound healing. In the present communication, it can be surmised that the simultaneous application of NPWT and micrografts has a clinically favorable effect on the rapid formation of granulation tissue; NPWT may ameliorate exudative wounds to a better circumstance for cells, and micrografted tissues may initiate granulation tissue developments. To our knowledge, no article has been reported in a clinical case where a refractory cutaneous ulcer lesion was managed with both NPWT and micrografts at the same time. Therefore, our concern was following micrografts infiltration when and with what pressure NPWT should be re-started. In our protocol, the micrografts suspension was injected several millimeters in depth around and over the wound surface, and the NPWT device was applied at −125 mmHg on the same day. Administration of negative pressure on the lesion after the engraftment of micronized autologous tissue could potentially suck out the micrografts, which, in turn, may possibly scale down the wound healing ability that micrografts possess. It is of paramount importance to maintain the tissue amount grafted in a lesion to initiate active and substantial healing. 

## 5. Conclusions

By combining the NPWT and autologous dermal micrografts, we were able to achieve additional benefits to the wound healing which was clinically confirmed through rapidly accelerated granulation formation in the patient’s lower leg at the chronic ulcer site. Moreover, it is conceivable that it may take several days of interval before resuming the NPWT after micrografting, or even applying it at a weaker negative pressure setting, such as at −5 mmHg. To clarify this point, further investigation would be necessary to identify the true potentials of this combination therapy.

## References

[1] Malmsjö M., Gustafsson L., Lindstedt S., Gesslein B., Ingemansson R. (2012). The effects of variable, intermittent, and continuous negative pressure wound therapy, using foam or gauze, on wound contraction, granulation tissue formation, and ingrowth into the wound filler. Eplasty.

[2] Shah A., Sumpio B.J., Tsay C., Swallow M., Dash B., Thorn S.L., Sinusas A.J., Koo A., Hsia H.C., Au A. (2019). Incisional Negative Pressure Wound Therapy Augments Perfusion and Improves Wound Healing in a Swine Model Pilot Study. Ann. Plast. Surg..

[3] Yang S.L., Zhu L.Y., Han R., Sun L.L., Dou J.T. (2017). Effect of Negative Pressure Wound Therapy on Cellular Fibronectin and Transforming Growth Factor-beta1 Expression in Diabetic Foot Wounds. Foot Ankle Int..

[4] Meek C.P. (1958). Successful microdermagrafting using the Meek-Wall microdermatome. Am. J. Surg..

[5] Klosová H., Němečková Crkvenjaš Z., Štětinský J. (2017). Meek micrografting technique and its use in the treatment of severe burn injuries at the University Hospital Ostrava burn center. Acta Chir. Plast..

[6] Trovato L., Monti M., Del Fante C., Cervio M., Lampinen M., Ambrosio L., Redi C.A., Perotti C., Kankuri E., Ambrosio G. (2015). A New Medical device rigeneracons allows to obtain viable micro-grafts from mechanical disaggregation of human tissues. J. Cell Physiol..

[7] Jimi S., Kimura M., De Francesco F., Riccio M., Hara S., Ohjimi H. (2017). Acceleration Mechanisms of Skin Wound Healing by Autologous Micrograft in Mice. Int. J. Mol. Sci..

[8] Jimi S., Takagi S., De Francesco F., Miyazaki M., Saparov A. (2020). Acceleration of Skin Wound-Healing Reactions by Autologous Micrograft Tissue Suspension. Medicina.

[9] Alvarez X., Valenzuela M., Tuffet J., Tuffet C. (2017). Microscopic and Histologic Evaluation of the Regenera Method for the Treatment of Androgenetic Alopecia in a Small Number of Cases. Int. J. Res. Stud. Med. Health Sci..

[10] Baglioni E., Trovato L., Marcarelli M., Frenello A., Bocchiotti M.A. (2016). Treatment of Oncological Post-surgical Wound Dehiscence with Autologous Skin Micrografts. Anticancer Res..

[11] Andreone A., Hollander D.D. (2019). A retrospective Study on the Use of Dermis Micrografts in Platelet-Rich Fibrin for the Resurfacing of Massive and Chronic Full-Thickness Burns. Stem Cells Int..

[12] De Francesco F., Mannucci S., Conti G., Pre E.D., Sbarbati A., Riccio M. (2018). A Non-Enzymatic Method to Obtain a Fat Tissue Derivative Highly Enriched in Adipose Stem Cells (ASCs) from Human Lipoaspirates: Preliminary Results. Int. J. Mol. Sci..

[13] D’Aquino R., Trovato L., Graziano A., Ceccarelli G., Cusella de Angelis G., Marangini A., Nisio A., Galli M., Pasi M., Finotti M. (2016). Periosteum-derived micro-grafts for tissue regeneration of human maxillary bone. J. Transl. Sci..

[14] Dorta Fernandez A., Baroni Luengo A. (2018). Biostimulation of Knee Cartilage Using Autologous Micro-Grafts: A Preliminary Study of the Rigenera Protocol in Osteochondral Lesions of the Knee. Rehabil. Sci..

[15] Marcarelli M., Fiammengo M., Trovato L., Lancione V., Novarese E., Indelli P.F., Risitano S. (2020). Autologous grafts in the treatment of avascular osteonecrosis of the femoral head. Acta Biomed..

[16] Xie Y., Lampinen M., Takala J., Sikorski V., Soliymani R., Tarkia M., Lalowski M., Mervaala E., Kupari M., Zheng Z. (2020). Epicardial transplantation of atrial appendage micrograft patch salvages myocardium after infarction. J. Heart Lung Transplant..

[17] Barchitta M., Maugeri A., Favara G., Magnano San Lio R., Evola G., Agodi A., Basile G. (2019). Nutrition and Wound Healing: An Overview Focusing on the Beneficial Effects of Curcumin. Int. J. Mol. Sci..

[18] De Francesco F., Graziano A., Trovato L., Ceccarelli G., Romano M., Marcarelli M., Cusella de Angelis G.M., Cillo U., Riccio M., Ferraro G.A. (2017). A Regenerative Approach with Dermal Micrografts in the Treatment of Chronic Ulcers. Stem Cell Rev..

[19] Miranda R., Farina E., Farina M.A. (2018). Micrografting chronic lower extremity ulcers with mechanically disaggregated skin using a micrograft preparation system. J. Wound Care.

[20] Svolacchia F., De Francesco F., Trovato L., Graziano A., Ferraro G.A. (2016). An innovative regenerative treatment of scars with dermal micrografts. J. Cosmet. Dermatol..

[21] Marcarelli M., Trovato L., Novarese E., Riccio M., Graziano A. (2017). Rigenera protocol in the treatment of surgical wound dehiscence. Int. Wound J..

[22] Riccio M., Marchesini A., Zingaretti N., Carella S., Senesi L., Onesti M.G., Parodi P.C., Ribuffo D., Vaienti L., De Francesco F. (2019). A Multicentre Study: The Use of Micrografts in the Reconstruction of Full-Thickness Posttraumatic Skin Defects of the Limbs-A Whole Innovative Concept in Regenerative Surgery. Stem Cells Int..

[23] Balli M., Vitali F., Janiszewski A., Caluwé E., Cortés-Calabuig A., Carpentier S., Duelen R., Ronzoni F., Marcelis L., Bosisio F.M. (2020). Autologous micrograft accelerates endogenous wound healing response through ERK-induced cell migration. Cell Death Differ..

[24] Ruiz R.G., Rosell J.M.C., Ceccarelli G., Ceccarelli G., de Sio C., de Angelis G.C., Pinto H., Astarita C., Graziano A. (2019). Progenitor-cell-enriched micrografts as a novel option for the management of androgenetic alopecia. J. Cell Physiol..

[25] Lian N., Li T. (2016). Growth factor pathways in hypertrophic scars: Molecular pathogenesis and therapeutic implications. Biomed. Pharmacother..

